# 4-Phenyl­piperazin-1-ium dihydrogen phosphate

**DOI:** 10.1107/S1600536810030813

**Published:** 2010-08-11

**Authors:** Manel Essid, Houda Marouani, Mohamed Rzaigui, Salem S. Al-Deyab

**Affiliations:** a Laboratoire de Chimie des Matériaux, Faculté des Sciences de Bizerte, 7021 Zarzouna Bizerte, Tunisia; b Petrochemical Research Chair, College of Science, King Saud University, Riadh, Saudi Arabia

## Abstract

The title compound, C_10_H_15_N_2_^+^·H_2_PO_4_^−^, is built up from 4-phenyl­piperazin-1-ium cations and dihydrogen phosphate anions. The inter­connection between two adjacent anions is assured by two strong O—H⋯O hydrogen bonds, which lead to the formation of infinite wave-like chains which spread along the *a* axis. The organic cations connect these chains *via* N—H⋯O hydrogen bonds. The crystal cohesion and stability are ensured by electrostatic and van der Waals inter­actions which, together with N—H⋯O and O—H⋯O hydrogen bonds, build up a two-dimensional network.

## Related literature

For the pharmacological properties of phenyl­piperazines and their derivatives, see: Cohen *et al.* (1982 ▶); Conrado *et al.* (2008 ▶); Neves *et al.* (2003 ▶); Hanano *et al.* (2000 ▶). For related structures, see: Zouari *et al.* (1995 ▶); Ben Gharbia *et al.* (2005 ▶). For a discussion of hydrogen bonding, see: Brown (1976 ▶); Blessing (1986 ▶). For tetra­hedral distortions, see: Baur (1974 ▶). For structural discussion, see: Ferraris & Ivaldi (1984 ▶); Janiak (2000 ▶).
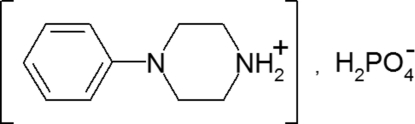


## Experimental

### 

#### Crystal data

C_10_H_15_N_2_^+^·H_2_PO_4_^−^
*M* *_r_* = 260.23
Orthorhombic, 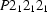

*a* = 6.175 (3) Å
*b* = 8.276 (3) Å
*c* = 24.408 (9) Å
*V* = 1247.3 (9) Å^3^
*Z* = 4
Ag *K*α radiation
λ = 0.56083 Å
μ = 0.12 mm^−1^
*T* = 293 K
0.50 × 0.40 × 0.10 mm

#### Data collection

Enraf–Nonius CAD-4 diffractometer
4505 measured reflections
4296 independent reflections
2134 reflections with *I* > 2σ(*I*)
*R*_int_ = 0.029
2 standard reflections every 120 min  intensity decay: 6%

#### Refinement

*R*[*F* ^2^ > 2σ(*F* ^2^)] = 0.056
*wR*(*F* ^2^) = 0.127
*S* = 0.94
4296 reflections
154 parameters
H-atom parameters constrained
Δρ_max_ = 0.44 e Å^−3^
Δρ_min_ = −0.27 e Å^−3^
Absolute structure: Flack (1983 ▶), 812 Friedel pairs
Flack parameter: −0.1 (2)

### 

Data collection: *CAD-4 EXPRESS* (Enraf–Nonius, 1994 ▶); cell refinement: *CAD-4 EXPRESS*; data reduction: *XCAD4* (Harms & Wocadlo, 1996 ▶); program(s) used to solve structure: *SHELXS97* (Sheldrick, 2008 ▶); program(s) used to refine structure: *SHELXL97* (Sheldrick, 2008 ▶); molecular graphics: *ORTEP-3 for Windows* (Farrugia, 1997 ▶) and *DIAMOND* (Brandenburg & Putz, 2005 ▶); software used to prepare material for publication: *WinGX* (Farrugia, 1999 ▶).

## Supplementary Material

Crystal structure: contains datablocks I, global. DOI: 10.1107/S1600536810030813/dn2592sup1.cif

Structure factors: contains datablocks I. DOI: 10.1107/S1600536810030813/dn2592Isup2.hkl

Additional supplementary materials:  crystallographic information; 3D view; checkCIF report

## Figures and Tables

**Table 1:** Hydrogen-bond geometry (Å, °)

*D*—H⋯*A*	*D*—H	H⋯*A*	*D*⋯*A*	*D*—H⋯*A*
O1—H1⋯O4^i^	0.82	1.81	2.587 (3)	158
O2—H2⋯O4^ii^	0.82	1.87	2.642 (3)	156
N1—H1*B*⋯O3	0.90	1.84	2.731 (3)	171
N1—H1*A*⋯O3^iii^	0.90	1.79	2.675 (3)	167

Symmetry codes: (i) 
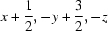
; (ii) 
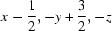
; (iii) 
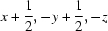
.

**Fig. 1.**
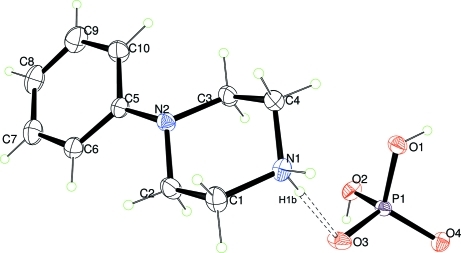
An ORTEP view of (I) with the atom-labelling scheme. Displacement ellipsoids are drawn at the 30% probability level. H atoms are represented as small spheres of arbitrary radii.

**Fig. 2.**
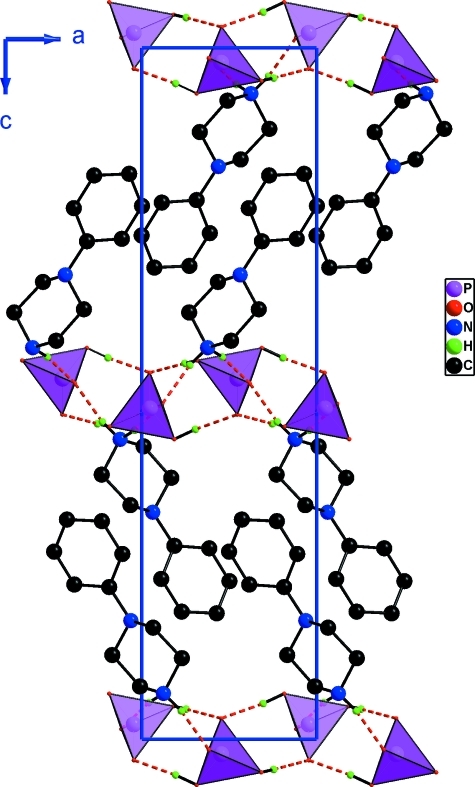
Projection of (I) along the b axis. H atoms non committed in H-bonds are omitted.

## supplementary crystallographic information

### Comment

Research relate to a novel group of phenylpiperazines and its derivatives having interesting pharmacological, cardiovascular and autonomic properties such as a high affinity for the dopamine D.sub.2 receptor and/or the serotonin reuptake site, and the ability to treat conditions related to disturbances in the dopaminergic and/or the serotonergic systems such as anxiety disorders, depression, Parkinson's disease, and schizophrenia (Conrado *et al.*, 2008); (Cohen *et al.*, 1982); (Neves *et al.*, 2003). In addition, novel phenylpiperazine derivatives were synthesized as dual cytokine regulators with TNF-alpha suppressing and IL-10 augmenting activity (Hanano *et al.*, 2000).

In this work, we report the preparation and the structural investigation of the noncentrosymmetric, (C_10_H_15_N_2_)H_2_PO_4_, (I). This compound is built up from the H_2_PO_4_^-^ anion and the organic 4-phenylpiperazin-1-ium cation (Fig. 1). The atomic arrangement can be described as a stacking of H_2_PO_4_^-^ anions according to the *a* axis, forming chains located in the planes *z* = 0 and *z* = 1/2. These chains are themselves interconnected by means of the N—H···O hydrogen bonds. Between which are located the organic cations. Examination of the H_2_PO_4_^-^ geometry shows two types of P—O distances. The largest ones, 1.567 (2) Å and 1.568 (5) Å, can be attributed to the P—OH distances, while the shortest ones, 1.511 (6) Å and 1.495 (9) Å, correspond to the phosphoric atom doubly bonded to the oxygen atom (P=O). The average values of the P—O distances and O—P—O angles are 1.533 Å and 109.4 °, respectively. These geometrical features have also been noticed in other crystal structures (Ferraris, *et al.*, 1984). Nevertheless, the calculated average values of the distortion indices (Baur, 1974). corresponding to the different angles and distances in the PO_4_ tetrahedron [DI(PO) = 0.019, DI(OPO) = 0.027, and DI(OO) = 0.014] show a slight distortion of the OPO angles if compared to O—O and PO distances. So, the PO_4_ group can be considered as a rigid regular arrangement of oxygen atoms, with the phosphorus atom slightly displaced from the gravity centre.

The interconnection between two adjacent anions H_2_PO_4_^-^ is assured by two strong H-bond [d (O···O) < 2.73 Å] (Brown, 1976); (Blessing, 1986) to form infinite waved chains which spread along the *a* axis.

The protonation of the phenylpiperazine can be due to the higher basicity and less constraint on this hydrogen. The piperazinium ring has a chair conformation, the most stable chemical conformation, with bond angles of around 109 °. The distances of the N atoms from the main plane through the carbon atoms of 0.64 and 0.63 Å. The interatomic bond lengths and angles of the organic groups spread respectively within the ranges [1.366 (6)–1.498 (5) Å] and [110.3 (2)–122.6 (3)°]. The aromatic rings are planar with an average deviation of 0.000343 Å show no significant deviation from those obtained in other 4-phenylpiperazin-1-ium salts such as [C_10_H_15_N_2_]HgCl_3_ (Zouari, *et al.*, 1995) and [C_10_H_16_N_2_]_2_ZnCl_4_ (Ben Gharbia, *et al.*, 2005). The phenylpiperazinium cations are organized in opposite direction along the *b* axis between the inorganic layers. Furthermore, the inorganic anion chains screen the interaction between the organic cations and probably lead to a non-centrosymmetric atomic arrangement. Therefore, the title compound could be an interesting material in the non-linear optics.

The interplanar distance between nearby phenyl rings is in the vicinity of 4.870 Å, which is significantly longer than 3.80 Å for the π-π interaction (Janiak, 2000). The organic cations are linked onto the anionic chains, by forming H-bonds with the oxygen atoms with N—H···O distances in the range 2.675 (3) - 2.731 (3) Å. These hydrogen bonds contribute to the cohesion and stability of the network of the studied crystal structure.

### Experimental

Single crystals of the title compound were prepared at room temperature from a mixture of an aqueous solution of phosphoric acid (2 mmol), 1-phenylpiperazine (2 mmol), ethanol (10 ml) and water (10 ml). The resulting solution was stirred during 1 h then evaporated slowly at room temperature for several days until the formation of good quality of prismatic single crystals.

### Refinement

All H atoms attached to C atoms and N atom were fixed geometrically and treated as riding with C—H = 0.93 Å (aromatic) or 0.97 Å (methylene) and N—H = 0.90 Å with U_iso_(H) = 1.2U_eq_(C or N).

Owing to the low number of Friedel pairs, the standard deviation on the Flack parameter is large, -0.1 (2). However inverting the structure lead to a value of 1.1 (2) and then it was assumed that the correct absolute structure corresponds to the refined model.

### Crystal data

**Table:**

C_10_H_15_N_2_^+^·H_2_PO_4_^−^	*F*(000) = 552
*M**_r_* = 260.23	*D*_x_ = 1.386 Mg m^−^^3^
Orthorhombic, *P*2_1_2_1_2_1_	Ag *K*α radiation, λ = 0.56083 Å
Hall symbol: P 2ac 2ab	Cell parameters from 25 reflections
*a* = 6.175 (3) Å	θ = 9–11°
*b* = 8.276 (3) Å	µ = 0.12 mm^−^^1^
*c* = 24.408 (9) Å	*T* = 293 K
*V* = 1247.3 (9) Å^3^	Prism, colourless
*Z* = 4	0.50 × 0.40 × 0.10 mm

### Data collection

**Table:**

Enraf–Nonius CAD-4 diffractometer	*R*_int_ = 0.029
Radiation source: Enraf Nonius FR590	θ_max_ = 28.0°, θ_min_ = 2.6°
graphite	*h* = 0→10
non–profiled ω scans	*k* = 0→13
4505 measured reflections	*l* = −10→40
4296 independent reflections	2 standard reflections every 120 min
2134 reflections with *I* > 2σ(*I*)	intensity decay: 6%

### Refinement

**Table:**

Refinement on *F*^2^	Secondary atom site location: difference Fourier map
Least-squares matrix: full	Hydrogen site location: inferred from neighbouring sites
*R*[*F*^2^ > 2σ(*F*^2^)] = 0.056	H-atom parameters constrained
*wR*(*F*^2^) = 0.127	*w* = 1/[σ^2^(*F*_o_^2^) + (0.0539*P*)^2^] where *P* = (*F*_o_^2^ + 2*F*_c_^2^)/3
*S* = 0.94	(Δ/σ)_max_ < 0.001
4296 reflections	Δρ_max_ = 0.44 e Å^−^^3^
154 parameters	Δρ_min_ = −0.27 e Å^−^^3^
0 restraints	Absolute structure: Flack (1983), 812 Friedel pairs
Primary atom site location: structure-invariant direct methods	Flack parameter: −0.1 (2)

### Special details

**Table:**

Geometry. All esds (except the esd in the dihedral angle between two l.s. planes) are estimated using the full covariance matrix. The cell esds are taken into account individually in the estimation of esds in distances, angles and torsion angles; correlations between esds in cell parameters are only used when they are defined by crystal symmetry. An approximate (isotropic) treatment of cell esds is used for estimating esds involving l.s. planes.
Refinement. Refinement of *F*^2^ against ALL reflections. The weighted *R*-factor *wR* and goodness of fit *S* are based on *F*^2^, conventional *R*-factors *R* are based on *F*, with *F* set to zero for negative *F*^2^. The threshold expression of *F*^2^ > σ(*F*^2^) is used only for calculating *R*-factors(gt) *etc*. and is not relevant to the choice of reflections for refinement. *R*-factors based on *F*^2^ are statistically about twice as large as those based on *F*, and *R*- factors based on ALL data will be even larger.

### Fractional atomic coordinates and isotropic or equivalent isotropic displacement parameters (Å^2^)

**Table:**

	*x*	*y*	*z*	*U*_iso_*/*U*_eq_	
C1	0.9900 (5)	0.0405 (3)	0.07656 (13)	0.0538 (8)	
H1C	1.0834	−0.0497	0.0858	0.065*	
H1D	0.9118	0.0128	0.0434	0.065*	
C2	0.8320 (5)	0.0668 (4)	0.12197 (12)	0.0540 (8)	
H2A	0.7262	0.1468	0.1107	0.065*	
H2B	0.7557	−0.0333	0.1293	0.065*	
C3	1.0653 (6)	0.2676 (3)	0.16155 (12)	0.0529 (7)	
H3A	1.1387	0.2996	0.1950	0.063*	
H3B	0.9697	0.3549	0.1507	0.063*	
C4	1.2291 (5)	0.2386 (4)	0.11736 (13)	0.0525 (8)	
H4A	1.3089	0.3375	0.1105	0.063*	
H4B	1.3313	0.1570	0.1294	0.063*	
C5	0.8136 (4)	0.1192 (3)	0.21995 (12)	0.0413 (6)	
C6	0.6143 (5)	0.0404 (4)	0.22358 (14)	0.0516 (8)	
H6	0.5592	−0.0117	0.1929	0.062*	
C7	0.4984 (6)	0.0382 (4)	0.27123 (16)	0.0647 (10)	
H7	0.3657	−0.0149	0.2721	0.078*	
C8	0.5724 (7)	0.1121 (4)	0.31782 (16)	0.0705 (10)	
H8	0.4907	0.1116	0.3498	0.085*	
C9	0.7710 (8)	0.1870 (5)	0.31582 (15)	0.0769 (12)	
H9	0.8261	0.2352	0.3473	0.092*	
C10	0.8895 (6)	0.1921 (4)	0.26826 (13)	0.0588 (8)	
H10	1.0225	0.2448	0.2680	0.071*	
N1	1.1243 (4)	0.1849 (3)	0.06624 (10)	0.0396 (5)	
H1A	1.2262	0.1615	0.0411	0.047*	
H1B	1.0409	0.2649	0.0529	0.047*	
N2	0.9374 (4)	0.1210 (2)	0.17176 (9)	0.0406 (5)	
P1	0.95373 (11)	0.58755 (7)	0.02154 (3)	0.03303 (15)	
O1	1.1831 (3)	0.5806 (2)	0.04795 (8)	0.0458 (5)	
H1	1.2422	0.6688	0.0448	0.069*	
O2	0.8135 (3)	0.6752 (3)	0.06613 (8)	0.0484 (5)	
H2	0.6912	0.6906	0.0542	0.073*	
O3	0.8824 (3)	0.4159 (2)	0.01488 (9)	0.0521 (5)	
O4	0.9544 (3)	0.6870 (2)	−0.03041 (7)	0.0418 (4)	

### Atomic displacement parameters (Å^2^)

**Table:**

	*U*^11^	*U*^22^	*U*^33^	*U*^12^	*U*^13^	*U*^23^
C1	0.069 (2)	0.0385 (12)	0.0542 (18)	−0.0065 (13)	0.0118 (16)	−0.0106 (12)
C2	0.0533 (16)	0.0602 (18)	0.0485 (16)	−0.0178 (16)	0.0073 (14)	−0.0121 (15)
C3	0.0628 (17)	0.0529 (15)	0.0428 (15)	−0.0225 (17)	0.0014 (16)	−0.0069 (13)
C4	0.0454 (15)	0.0566 (17)	0.0555 (18)	−0.0096 (14)	−0.0052 (15)	0.0093 (15)
C5	0.0455 (14)	0.0342 (13)	0.0441 (15)	0.0061 (10)	0.0024 (13)	0.0034 (11)
C6	0.0549 (17)	0.0519 (17)	0.0480 (17)	−0.0019 (13)	0.0034 (15)	0.0042 (14)
C7	0.061 (2)	0.066 (2)	0.067 (2)	0.0014 (15)	0.0155 (19)	0.0143 (18)
C8	0.091 (3)	0.0611 (19)	0.059 (2)	0.017 (2)	0.028 (2)	0.0121 (18)
C9	0.123 (4)	0.062 (2)	0.0452 (19)	−0.005 (3)	0.008 (2)	−0.0109 (17)
C10	0.075 (2)	0.0543 (16)	0.0471 (17)	−0.0141 (16)	0.0025 (17)	−0.0121 (15)
N1	0.0432 (11)	0.0319 (9)	0.0436 (12)	0.0102 (9)	0.0062 (11)	0.0050 (10)
N2	0.0445 (11)	0.0364 (10)	0.0410 (12)	−0.0047 (10)	0.0008 (11)	−0.0046 (9)
P1	0.0347 (3)	0.0253 (2)	0.0391 (3)	−0.0019 (3)	−0.0063 (3)	0.0030 (3)
O1	0.0424 (9)	0.0369 (9)	0.0582 (12)	−0.0069 (9)	−0.0132 (9)	0.0143 (9)
O2	0.0507 (11)	0.0541 (11)	0.0403 (10)	0.0097 (10)	0.0004 (9)	0.0037 (9)
O3	0.0599 (10)	0.0271 (7)	0.0693 (13)	−0.0028 (8)	−0.0286 (11)	0.0008 (10)
O4	0.0392 (8)	0.0443 (8)	0.0418 (10)	0.0089 (9)	0.0007 (9)	0.0084 (8)

### Geometric parameters (Å, °)

**Table:**

C1—N1	1.476 (4)	C6—C7	1.366 (5)
C1—C2	1.492 (4)	C6—H6	0.9300
C1—H1C	0.9700	C7—C8	1.370 (6)
C1—H1D	0.9700	C7—H7	0.9300
C2—N2	1.450 (4)	C8—C9	1.375 (6)
C2—H2A	0.9700	C8—H8	0.9300
C2—H2B	0.9700	C9—C10	1.373 (5)
C3—N2	1.469 (3)	C9—H9	0.9300
C3—C4	1.498 (5)	C10—H10	0.9300
C3—H3A	0.9700	N1—H1A	0.9000
C3—H3B	0.9700	N1—H1B	0.8998
C4—N1	1.474 (4)	P1—O3	1.496 (2)
C4—H4A	0.9700	P1—O4	1.5114 (19)
C4—H4B	0.9700	P1—O1	1.5572 (19)
C5—C6	1.395 (4)	P1—O2	1.569 (2)
C5—N2	1.403 (4)	O1—H1	0.8197
C5—C10	1.405 (4)	O2—H2	0.8194
			
N1—C1—C2	112.1 (2)	C6—C7—C8	121.7 (3)
N1—C1—H1C	109.2	C6—C7—H7	119.1
C2—C1—H1C	109.2	C8—C7—H7	119.1
N1—C1—H1D	109.2	C7—C8—C9	118.0 (3)
C2—C1—H1D	109.2	C7—C8—H8	121.0
H1C—C1—H1D	107.9	C9—C8—H8	121.0
N2—C2—C1	112.0 (2)	C10—C9—C8	121.3 (4)
N2—C2—H2A	109.2	C10—C9—H9	119.4
C1—C2—H2A	109.2	C8—C9—H9	119.4
N2—C2—H2B	109.2	C9—C10—C5	121.2 (3)
C1—C2—H2B	109.2	C9—C10—H10	119.4
H2A—C2—H2B	107.9	C5—C10—H10	119.4
N2—C3—C4	110.7 (2)	C4—N1—C1	110.3 (2)
N2—C3—H3A	109.5	C4—N1—H1A	109.6
C4—C3—H3A	109.5	C1—N1—H1A	109.6
N2—C3—H3B	109.5	C4—N1—H1B	109.6
C4—C3—H3B	109.5	C1—N1—H1B	109.6
H3A—C3—H3B	108.1	H1A—N1—H1B	108.2
N1—C4—C3	111.2 (2)	C5—N2—C2	117.0 (2)
N1—C4—H4A	109.4	C5—N2—C3	116.4 (2)
C3—C4—H4A	109.4	C2—N2—C3	110.8 (2)
N1—C4—H4B	109.4	O3—P1—O4	115.26 (11)
C3—C4—H4B	109.4	O3—P1—O1	106.11 (11)
H4A—C4—H4B	108.0	O4—P1—O1	111.40 (11)
C6—C5—N2	122.6 (3)	O3—P1—O2	110.60 (12)
C6—C5—C10	116.2 (3)	O4—P1—O2	109.38 (11)
N2—C5—C10	121.1 (3)	O1—P1—O2	103.41 (12)
C7—C6—C5	121.5 (3)	P1—O1—H1	109.5
C7—C6—H6	119.2	P1—O2—H2	109.5
C5—C6—H6	119.2		

### Hydrogen-bond geometry (Å, °)

**Table:**

*D*—H···*A*	*D*—H	H···*A*	*D*···*A*	*D*—H···*A*
O1—H1···O4^i^	0.82	1.81	2.587 (3)	158
O2—H2···O4^ii^	0.82	1.87	2.642 (3)	156
N1—H1B···O3	0.90	1.84	2.731 (3)	171
N1—H1A···O3^iii^	0.90	1.79	2.675 (3)	167

Symmetry codes: (i) *x*+1/2, −*y*+3/2, −*z*; (ii) *x*−1/2, −*y*+3/2, −*z*; (iii) *x*+1/2, −*y*+1/2, −*z*.
